# Efficacy and Safety of Acotiamide for the Treatment of Functional Dyspepsia: Systematic Review and Meta-Analysis

**DOI:** 10.1155/2014/541950

**Published:** 2014-08-12

**Authors:** Guoguang Xiao, Xiaoping Xie, Juan Fan, Jianjun Deng, Shan Tan, Yu Zhu, Qin Guo, Chaomin Wan

**Affiliations:** ^1^Department of Pediatrics, West China Second Hospital, Sichuan University, No. 20, Section 3, Renmin Nanlu, Chengdu, Sichuan 610041, China; ^2^Department of Pediatrics, The Medical Center of Dujiangyan, Chengdu 611830, China; ^3^Department of Pediatrics, Sichuan Academy of Medical Science and Sichuan Provincial People's Hospital, Chengdu 610072, China

## Abstract

*Background.* There are no treatments with established efficacy for this disorder so far. *Aim.* To systematically review the efficacy of acotiamide in the treatment of patients with FD. *Methods.* We searched main electronic databases through November 2013. RCTs evaluating the efficacy of acotiamide versus placebo in FD patients were included. Pooled risk ratio (RR) with 95% confidential interval (CI) was calculated. *Results.* Six publications including seven RCTs were eligible for inclusion. The summary RR of overall improvement of FD symptoms in patients receiving acotiamide versus placebo was 1.29 (95% CI, 1.19–1.40, *P* < 0.00001; *I* ^2^ = 15%). Acotiamide improved the symptoms of patients with postprandial distress syndrome (PDS) (RR, 1.29; 95% CI, 1.09–1.53, *P* = 0.003; *I* ^2^ = 0%), and the summary RR for patients with epigastric pain syndrome (EPS) was 0.92 (95% CI, 0.76–1.11, *P* = 0.39; *I* ^2^ = 0%). Acotiamide showed a significantly beneficial effect on the elimination of some individual FD symptoms compared with placebo. Adverse events were not significantly different between acotiamide and placebo groups. Subgroup analyses suggested that acotiamide 100 mg three times daily (tid) showed consistent efficacy not only for the overall improvement but also for the elimination of some individual symptoms in FD patients. *Conclusions.* Acotiamide has the potential to improve the symptoms of patients with FD, particularly of patients with PDS, without major adverse effects. The dosage of acotiamide 100 mg tid might be the appropriate dose in the treatment of FD.

## 1. Introduction

Functional dyspepsia (FD) is a clinical syndrome defined as the presence of symptoms thought to originate from the gastroduodenal region in the absence of organic, systemic, or metabolic disease likely to explain the symptoms [1]. FD is a common morbid condition and has great impact on the quality of life, healthcare usage, and socioeconomic costs [2]. The underlying pathophysiology of FD is incompletely understood and may be heterogeneous, possibly including mechanisms such as hypersensitivity to gastric distension [3], impaired meal accommodation [4, 5], and delayed gastric emptying [6, 7]. According to the Rome III consensus, the main symptoms of FD include bothersome postprandial fullness, early satiety, epigastralgia, and epigastric burning, and FD is further subdivided into two subcategories: meal-induced postprandial distress syndrome (PDS, characterized by postprandial fullness and early satiation) and epigastric pain syndrome (EPS, characterized by epigastric pain and burning) [1].

The primary treatment options to FD may include gastric acid suppression and gastroduodenal prokinetic agents, and some other drugs may also be applied. The Rome III consensus suggested that EPS might respond to the acid-suppressive or antacid therapy and PDS might respond to the prokinetic or motility-modifying treatment [1]. Prokinetic drugs are a class of medications which can enhance gastric contractility through action on different types of receptors [8]. Some prokinetics such as cisapride and tegaserod were withdrawn from the market for the cardiovascular adverse effects [9]. To date, although several therapeutic methods have been evaluated in the treatment of FD, treatments with established efficacy for patients with FD are still limited.

Acotiamide is a new prokinetic agent which exerts its gastroprokinetic activity by enhancement of acetylcholine release via acting as an antagonist on the M1 and M2 muscarinic receptors in the enteric nervous system and inhibiting acetylcholinesterase activity [1, 10, 11]. Furthermore, it may also act directly on the gut and indirectly on the central nervous system by way of the brain-gut axis [12]. Studies have shown that acotiamide could enhance gastric emptying and gastric accommodation [10, 11, 13, 14]. Clinical studies have demonstrated that acotiamide may have no significant influence on gastric emptying rate or nutrient tolerance in healthy subjects [15]. And beneficial effects of acotiamide have been found in patients with FD, particularly with meal-related FD symptoms such as postprandial fullness, upper abdominal bloating, and/or early satiety [16–18]. Therefore, acotiamide could be a promising agent in the treatment of patients with FD.

This study was conducted to summarize the current evidence and systematically and critically evaluate the efficacy as well as its adverse effects of acotiamide in the treatment of patients with FD.

## 2. Materials and Methods

### 2.1. Search Strategy

MEDLINE, EMBASE, the Cochrane Central Register of Controlled Trials, BIOSIS Previews,* International Pharmaceutical Abstracts*, and the Clinicaltrials.gov website were searched from inception through November 2013 without any language restrictions. The search terms used included nonulcer, dyspepsia, functional dyspepsia, NUD, indigestion, acotiamide, Z-338, YM443, prokinetic drug, and randomized controlled trials. Roots and variants of these terms were also used in the database searching. The reference lists of relevant review articles and original reports were also searched to retrieve additional studies not identified by the database search.

### 2.2. Study Selection

Randomized controlled trials (RCTs) evaluating the efficacy of acotiamide compared with placebo in patients with FD were included. Duplicate publications or studies in which raw data of interest cannot be extracted were excluded. Two reviewers screened the identified studies for eligibility independently, with disagreements resolved by consensus.

### 2.3. Data Extraction and Quality Assessment

Data of eligible studies were extracted according to the intention-to-treat criteria by two reviewers independently using a standardized data collection form, and differences were resolved by discussion. If necessary, a third reviewer was consulted to reach consensus. Trial investigators were requested for additional unpublished data as needed. Data extracted included study design, publication details, patient characteristics (number of patients, age, gender, and dyspepsia symptoms), diagnostic criteria, treatment regimen (dose and duration), outcome measures, and study outcomes.

The quality of each eligible study was assessed by two reviewers separately using a modified Jadad scoring system [19, 20], with discrepancies reconciled by a third reviewer. A Jadad score of 5 indicates high quality and a score of 0 suggests poor quality of the included study.

### 2.4. Outcome Assessed

The primary endpoint was to assess the efficacy of acotiamide compared with placebo for the overall improvement of FD symptoms. Secondary endpoints included evaluating the efficacy of acotiamide for the treatment of PDS and EPS and for the elimination of individual FD symptoms when possible. Adverse events which might be related to the therapy were evaluated as well. Subgroup analyses based on the different doses of acotiamide were conducted to explore the potentially optimal dose of acotiamide in the treatment of FD.

### 2.5. Statistical Analysis

To estimate the efficacy of intervention, pooled risk ratio (RR) together with 95% confidential interval (CI) was calculated by the Mantel-Haenszel fixed-effect model if no significant heterogeneity was detected. When significant heterogeneity existed, a random-effects model was used to calculate the summary RR with 95% CI. Heterogeneity among studies was assessed using Cochran's *Q* test and Higgins' *I* ^2^ statistic, and it was considered significant when the *P* value was less than 0.10 [21] or the *I* ^2^ statistic was greater than 50% [22]. Publication bias was evaluated by inspecting funnel plot and statistically by Egger's test [23]. Sensitivity analysis was performed to evaluate whether the magnitude or the statistical result of the pooled estimate was changed after exclusion of any single study. *P* value less than 0.05 was considered statistically significant for all tests except for heterogeneity. Review Manager (RevMan version 5.2.5, Nordic Cochrane Centre, Copenhagen, Denmark) and STATA (version 12.0, StataCorp, College Station, TX, USA) were used for statistical analysis.

## 3. Results

**Figure 1:**
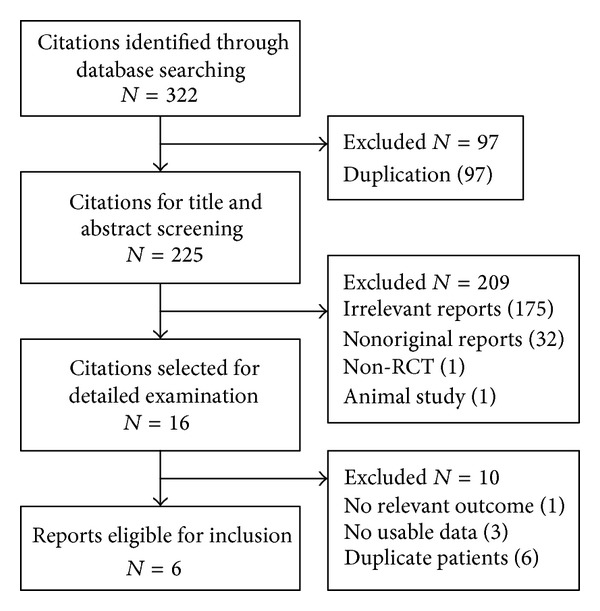
Flow diagram of study identification and selection.

The study selection process is summarized in Figure 1. The main database search identified a total of 225 citations and meeting abstracts after exclusion of duplication. Search within the Clinicaltrials.gov web site identified 8 records, all except one ongoing study (ClinicalTrials.gov NCT01973790) of which had been published as full text or meeting abstract in the journals and identified by our database search. Sixteen citations were selected for detailed examination. Among these citations, 10 meeting abstracts were excluded. Eventually, 4 articles published as full text and 2 meeting abstracts met the inclusion criteria and were included in this study [11, 16–18, 24, 25]. The authors of two publications [16, 25] reporting 3 RCTs provided us with some unpublished data about their studies. No eligible non-English studies were identified.

One publication reported two RCTs [16], and therefore 7 RCTs were included in our study. Among them, 5 RCTs were dose-ranging trial. The main characteristics of the 7 trials are summarized in Table 1. All trials were performed in a placebo-controlled, double-blind fashion. The mean patient age ranged from 37 to 49 years, whereas the data were not reported in the two meeting abstracts. The dose of oral acotiamide used ranged from 50 mg to 900 mg three times daily (tid). The duration of treatment was 3 to 4 weeks in most trials. The length of follow-up period was 4 weeks in two trials [17, 25] and unspecified in the remaining. The subjects' global assessment of overall treatment efficacy (OTE) was used to evaluate the efficacy of oral acotiamide for the overall improvement of subjective symptoms as the primary endpoint in 5 trials [11, 16, 18, 25], and patient's global symptomatic improvement (PGSI) was used in 1 trial [24]. Two studies investigated the underlying mechanisms as well as the efficacy and safety aspects of acotiamide in FD patients [11, 17]. The methodological quality of the included studies was good or excellent except the meeting abstracts included.

### 3.1. Overall Improvement of FD Symptoms

**Figure 2:**
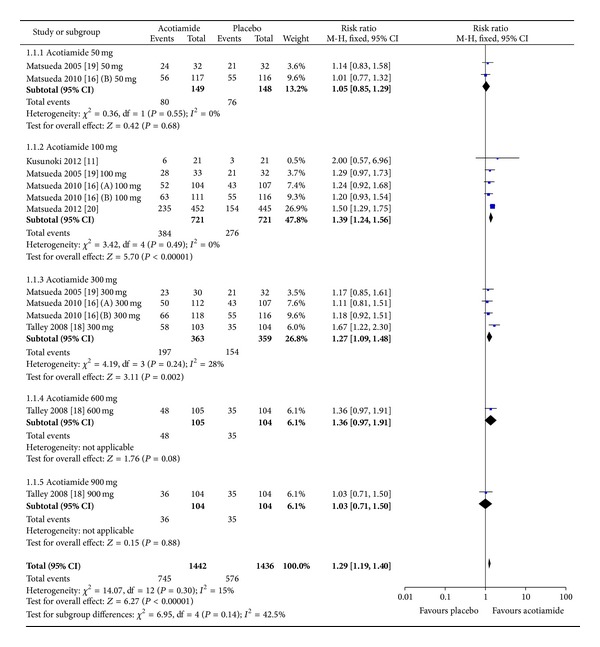
Pooled RR of overall improvement of FD symptoms in patients receiving acotiamide versus placebo.

**Figure 3:**
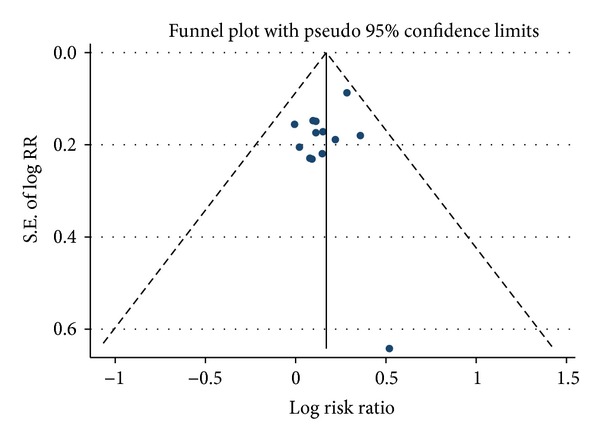
Funnel plot analysis. Funnel plot and Egger's test showed no evidence of publication bias.

A total of 6 trials reported the overall improvement of FD symptoms using OTE or PGSI as the endpoint, consisting of 2267 FD patients (acotiamide, 1442; placebo, 825) [11, 16, 18, 24, 25]. The pooled RR of the overall improvement of FD symptoms for the acotiamide groups versus placebo groups was 1.29 (95% CI, 1.19–1.40, *P* < 0.001; *I* ^2^ = 15%) using the fixed-effect model, suggesting that there was statistically significant improvement of symptoms within patients treated with acotiamide compared with those given placebo, as shown in Figure 2. Sensitivity analysis excluding 2 meeting abstracts with moderate methodological quality resulted in a pooled RR of 1.29 (95% CI, 1.17–1.42, *P* < 0.001; *I* ^2^ = 34%). When the study by Matsueda et al. [25] was excluded, the pooled RR was 1.21 (95% CI, 1.10–1.33, *P* < 0.001; *I* ^2^ = 0%) without statistical heterogeneity, suggesting that this study may be responsible for the previous heterogeneity. No significant publication bias was detected by funnel plot (Figure 3) or Egger's test (*P* = 0.347).

Subgroup analyses of different doses of acotiamide showed that the 100 mg group significantly improved the symptoms of the acotiamide group compared with the placebo group without significant heterogeneity (RR, 1.39; 95% CI, 1.24–1.56, *P* < 0.001; *I* ^2^ = 0%). Within the subgroup of acotiamide 300 mg, the pooled RR was 1.27 (95% CI, 1.09–1.48, *P* = 0.002; *I* ^2^ = 28%) with moderate heterogeneity and the pooled RR was 1.15 (95% CI, 0.97–1.37, *P* = 0.100; *I* ^2^ = 0%) without significant heterogeneity after exclusion of the included abstract by Talley et al. [18]. The summary efficacy was not significantly different in the remaining subgroups of acotiamide 50 mg, 600 mg, and 900 mg compared with the placebo group.

### 3.2. Efficacy of Acotiamide in PDS and EPS

Pooled analysis of the efficacy of acotiamide in the improvement of PDS yielded a summary RR of 1.29 (95% CI, 1.09–1.53, *P* = 0.003; *I* ^2^ = 0%), as shown in Table 2. As to EPS, the summary RR was 0.92 (95% CI, 0.76–1.11, *P* = 0.390; *I* ^2^ = 0%), as shown in Table 2. In the subgroup analyses of different doses of acotiamide, there was statistically significant efficacy in patients with PDS in the subgroup of acotiamide 100 mg (RR, 1.41; 95% CI, 1.07–1.85, *P* = 0.010; *I* ^2^ = 0%) and borderline efficacy in the 300 mg subgroup (RR, 1.33; 95% CI, 1.01–1.75, *P* = 0.040; *I* ^2^ = 0%) compared with the placebo group; no significant efficacy was observed within the three subgroups in the treatment of patients with EPS.

As shown in Table 3, there were no significant differences between the acotiamide and the placebo groups as to the overall symptoms, PDS, or EPS among patients without response to these treatments, except for the group receiving acotiamide 100 mg as to PDS, in which there seemed to be a lower rate of nonrespond compared with the placebo.

### 3.3. Elimination of Individual FD Symptoms

The pooled elimination efficacy of acotiamide in patients with postprandial fullness, upper abdominal bloating, and early satiety are presented in Table 2. The treatment of acotiamide showed a significantly positive effect on the elimination of individual FD symptoms including postprandial fullness, upper abdominal bloating, and early satiety, respectively, compared with the placebo group. Subgroup analyses suggested that the dose of acotiamide 100 mg showed a consistent efficacy for the elimination of the above three FD symptoms, although significant heterogeneity was detected in the analysis of postprandial fullness.

### 3.4. Adverse Events

The pooled estimates of common adverse events reported in the included trials are presented in Table 4. As can be seen in the table, adverse events listed were not significantly different between the acotiamide and placebo groups. Some other adverse events such as increase of blood potassium, lactic acid dehydrogenase, urine glucose and protein positivity, and dizziness and headache were reported but not commonly observed in the included trials. Two cases of serious adverse events, biliary colic and angina pectoris, were observed in patients treated with acotiamide 50 mg tid by Tack et al. [17] but were considered as unrelated or unlikely related to the trial. Another serious adverse events (intervertebral disc herniation) occurred in one patient treated with acotiamide and was judged as “not related” to the drug in the study by Matsueda et al. [25]. Four trials [16, 17, 24, 25] evaluated the electrocardiography of patients, without reporting notable abnormality in this aspect.

## 4. Discussion

This systematic review and meta-analysis has investigated the efficacy as well as adverse effects of a new drug, acotiamide, in the treatment of patients with FD. The results indicated that acotiamide could improve the FD symptoms, mainly symptoms of patients with PDS, compared with the placebo, with a quantitatively small but statistically significant summary RR compared with the placebo. Acotiamide was well tolerated during therapy without significantly increasing the adverse events in the studies reporting these data.

The OTE and PGSI assessments were used to evaluate the overall efficacy of acotiamide for relief of FD symptoms in some included studies [11, 16, 18, 24, 25]. And the result of overall improvement of FD symptoms after acotiamide treatment compared with the placebo from our meta-analysis was based on the OTE and PGSI assessments. These assessment endpoints are evaluated within the patients' own reference system and allow patients themselves to integrate all aspects of their conditions into a single therapeutic outcome [26]. And the advantage of these endpoints is that they closely resemble the way in which the physicians evaluate the therapeutic benefit in clinical practice [25]. Many other therapeutic studies in FD also used the OTE as an outcome measure [27–30].

The beneficial effect of acotiamide in patients with FD was also supported by other efficacy endpoints. Quality of life of FD patients treated with acotiamide was assessed by the SF-36 questionnaire [17, 18] and the Short Form-Nepean Dyspepsia Index questionnaire (SF-NDI) [18, 25]. The subscale of physical function on SF-36 was significantly improved in the acotiamide 100 mg group [17] and mental health on the SF-36 was significantly improved in all the 300 mg, 600 mg, and 900 mg groups compared with the placebo group [18]. The overall and subscale SF-NDI scores all showed significant improvement from baseline in the acotiamide 100 mg group compared with the placebo group [25]. And another study reported that treatment with acotiamide 300 mg significantly improved 3 of 5 SF-NDI subscales (tension, interference with daily life, and knowledge/control) [18]. Posttreatment follow-up study by Matsueda et al. [25] showed that the beneficial effect of acotiamide persisted during all 4-week follow-up period.

The results of our meta-analysis showed that acotiamide could be effective in the treatment of patients with PDS. And the elimination rates of three individual cardinal symptoms of PDS including postprandial fullness, upper abdominal bloating, and early satiety were significantly higher in the acotiamide groups than that in the placebo groups. The pathophysiological mechanisms associated with these PDS symptoms are mainly delayed gastric emptying and impaired gastric accommodation [4–7, 31, 32]. And acotiamide has been shown in the mechanistic studies to enhance the gastric emptying rate and gastric accommodation in FD patients [10, 11, 17], whereas no effect of acotiamide on gastric emptying was found within healthy volunteers [15]. However, not all PDS patients receiving acotiamide therapy get symptom relief, probably reflecting the heterogeneous property of this clinical condition [33]. Acotiamide did not significantly improved the symptoms of patients with EPS compared with placebo, possibly for the reason that the pathophysiological mechanisms underlying EPS are different from that of PDS and probably other management approaches are needed for EPS. Nevertheless, it has recently been demonstrated that there is overlap between PDS and EPS [9], suggesting that sometimes it may be difficult to distinguish these two subtypes of FD in the clinical practice.

The subgroup analyses of our study suggested that the dose of acotiamide 100 mg tid showed a consistent efficacy not only for the overall improvement of FD symptoms but also for the elimination of individual cardinal symptoms of PDS in FD patients. The relatively high improvement rate of FD symptoms by acotiamide 100 mg tid found in a multicenter, single-arm, long-term (48 weeks), phase III study [34] was similar to that observed in another 4-week phase III RCT [25]. These results indicate that the dose of 100 mg tid may be the optimal dosage of acotiamide in the treatment of FD. On the other hand, there was no apparent dose-response relationship between different dosages of acotiamide and the improvement of FD symptoms.

The incidences of adverse events were not significantly different between the acotiamide groups and placebo groups in our analysis. Acotiamide was well tolerated, and most adverse events reported in the included studies were mild or moderate. No clinical changes in vital signs or electrocardiographic variables were reported. Acotiamide has no affinity for the hERG channel [10]. No significant arrhythmogenic potential was found in the QTc studies with acotiamide up to 900 mg in the USA [25]. The single-arm, long-term (48 weeks), phase III trial also demonstrated a good safety profile of acotiamide in 409 FD patients, with one case of increased alanine aminotransferase as serious drug reaction [34].

There were several limitations to our meta-analysis, which need to be taken seriously when interpreting the results from this study. First, the present study possesses the inherent limitations associated with the methods of meta-analysis. Second, although the endpoints used in the included studies such as OTE and PGSI have the advantages mentioned above, these endpoints require patients' recall of the pretreatment symptom severity, which may induce bias. And aggravation of one or more specific symptoms may not be adequately identified by an overall endpoint [35]. Therefore, we evaluated the efficacy of acotiamide not only for the overall improvement of FD symptoms, but also for the elimination of three main symptoms of FD separately. Third, no publication bias was found in our study by the funnel plot analysis and Egger's test, and trials registered in Clinicaltrials.gov have all been published except one ongoing study. We also included both published studies and meeting abstracts in our analysis to avoid publication bias. However, potential publication bias cannot be ruled out completely because most studies included in the analysis were supported by the pharmaceutical company who had designed this drug and reported positive results. As to the two meeting abstracts, they were all sponsored and reported trials involving relatively large patient populations in the abstract form more than five years ago. However, no full publications about these two trials were identified through our systematic search. This may be a source of publication bias. Nonetheless, the summary RR of overall improvement of FD symptoms for the acotiamide versus placebo groups did not change significantly after exclusion of the two meeting abstracts. In addition, as FD is a chronic relapsing condition, follow-up data after cessation of treatment is of great help to evaluate if the benefit of acotiamide can last after treatment is stopped, but only few studies have reported those data.

In summary, our study suggests that acotiamide has the potential to improve the symptoms of patients with FD, particularly of patients with PDS, with a quantitatively small but statistically significant summary RR compared with the placebo, without major adverse effects. The dosage of acotiamide 100 mg three times daily seems to be the appropriate dose in the treatment of FD.

As the absolute benefit of acotiamide over placebo seems to be modest although statistically significant, and no apparent dose-response relationship between different dosages of acotiamide and the improvement of FD symptoms was found, more convincing evidence is needed to establish the efficacy of acotiamide in the treatment of FD. And most studies investigating the efficacy of acotiamide have been conducted in Japan so far; more studies from other parts of the world could help to confirm the efficacy of acotiamide in other populations. Future researches are needed to also investigate the long-lasting beneficial effects of acotiamide to prevent the relapse of FD and the long-term safety and tolerability profiles in large-scale, high quality clinical trials. Factors predicting a good response to acotiamide in the individual patient and the most appropriate treatment length also need further investigation.

## Figures and Tables

**Table 1:** Main characteristics of eligible studies.

Author, year	Location, and number of centers	Publication type	Diagnostic criteria	Number of patients	Dose of acotiamide	Duration of treatment	Jadad score
Kusunoki et al. 2012 [11]	Japan, 1 site	Full text	Rome II criteria	42	100 mg, tid	14–18 d	4
Matsueda et al. 2010 (A) [16]	Japan, 33 sites	Full text	Rome II criteria	323	100 mg or 300 mg, tid	4 w	5
Matsueda et al. 2010 (B) [16]	Japan, 46 sites	Full text	Rome II criteria	462	50 mg, 100 mg or 300 mg, tid	4 w	5
Tack et al. 2009 [17]	Europe, 8 sites	Full text	Rome II criteria	71	50 mg, 100 mg or 300 mg, tid	3 w	4
Talley et al. 2008 [18]	USA, 59 sites	Meeting abstract	Rome II criteria	416	300 mg, 600 mg or 900 mg, tid	12 w	3
Matsueda et al. 2005 [24]	Japan, NR	Meeting abstract	Patients with FD symptoms and negative endoscopy	127	50 mg, 100 mg or 300 mg, tid	4 w	2
Matsueda et al. 2012 [25]	Japan, 67 sites	Full text	Rome III criteria	897	100 mg, tid	4 w	5

FD, functional dyspepsia; NR, not reported; RCT, randomized controlled trial; and tid, three times daily.

**Table 2:** Pooled RR of acotiamide versus placebo for elimination of FD symptoms by Mantel-Haenszel fixed-effect model.

Groups and subgroups	Number of trials [reference]	Number of patients	RR; 95% CI	*P* value	Test of heterogeneity	
					*P* value	*I* ^2^
PDS	2 [16]	396	1.29; 1.09–1.53	0.003	0.830	0%
50 mg, tid	1 [16]	151	1.08; 0.76–1.53	0.660	NA	NA
100 mg, tid	2 [16]	206	1.41; 1.07–1.85	0.010	0.860	0%
300 mg, tid	2 [16]	218	1.33; 1.01–1.75	0.040	0.960	0%
EPS	2 [16]	320	0.92; 0.76–1.11	0.390	0.970	0%
50 mg, tid	1 [16]	73	0.88; 0.58–1.34	0.550	NA	NA
100 mg, tid	2 [16]	184	0.96; 0.72–1.29	0.800	0.520	0%
300 mg, tid	2 [16]	192	0.90; 0.67–1.21	0.480	0.990	0%
Postprandial fullness^a^	3 [16, 25]	1438	1.90; 1.37–2.64	<0.001	0.030	60%
50 mg, tid	1 [16]	149	3.19; 1.36–7.44	0.007	NA	NA
100 mg, tid	3 [16, 25]	1190	1.75; 1.14–2.66	0.010	0.060	64%
300 mg, tid	2 [16]	337	2.05; 0.79–5.34	0.140	0.040	77%
Upper abdominal bloating	3 [16, 25]	1232	1.30; 1.12–1.50	<0.001	0.930	0%
50 mg, tid	1 [16]	135	1.26; 0.79–2.01	0.330	NA	NA
100 mg, tid	3 [16, 25]	1001	1.29; 1.08–1.54	0.005	0.550	0%
300 mg, tid	2 [16]	310	1.34; 0.99–1.80	0.060	0.730	0%
Early satiety	3 [16, 25]	1206	1.39; 1.19–1.61	<0.001	0.830	0%
50 mg, tid	1 [16]	133	1.60; 0.95–2.70	0.080	NA	NA
100 mg, tid	3 [16, 25]	1002	1.39; 1.16–1.67	<0.001	0.450	0%
300 mg, tid	2 [16]	285	1.29; 0.94–1.76	0.110	0.830	0%

^a^Mantel-Haenszel random-effects model.

CI, confidential interval; FD, functional dyspepsia; NA, not applicable; RR, risk ratio; and tid, three times daily.

**Table 3:** Pooled RR of acotiamide versus placebo for nonresponders by Mantel-Haenszel fixed-effect model.

Groups or subgroups	Number of trials [reference]	Number of patients	RR; 95% CI	*P* value	Test of heterogeneity	
					*P* value	*I* ^2^
Overall symptoms	2 [16]	768	0.95; 0.73–1.24	0.710	0.510	0%
50 mg, tid	1 [16]	227	1.46; 0.78–2.73	0.230	NA	NA
100 mg, tid	2 [16]	431	0.83; 0.55–1.26	0.380	0.420	0%
300 mg, tid	2 [16]	441	0.90; 0.60–1.34	0.590	0.590	0%
PDS	2 [16]	396	0.71; 0.50–1.02	0.060	0.300	18%
50 mg, tid	1 [16]	151	1.22; 0.64–2.34	0.540	NA	NA
100 mg, tid	2 [16]	206	0.51; 0.26–0.99	0.050	0.370	0%
300 mg, tid	2 [16]	218	0.61; 0.34–1.10	0.100	0.560	0%
EPS^a^	2 [16]	320	1.76; 0.90–3.45	0.100	0.190	35%
50 mg, tid	1 [16]	73	3.89; 0.46–33.17	0.210	NA	NA
100 mg, tid	2 [16]	184	1.91; 0.48–7.57	0.360	0.170	47%
300 mg, tid	2 [16]	192	2.31; 0.34–15.74	0.390	0.070	70%

^a^Mantel-Haenszel random-effects model.

CI, confidential interval; FD, functional dyspepsia; NA, not applicable; RR, risk ratio; and tid, three times daily.

**Table 4:** Pooled RR of adverse events in FD patients receiving acotiamide versus placebo by Mantel-Haenszel fixed-effect model.

Groups	Number of trials [reference]	Number of patients	RR; 95% CI	*P* value	Test of heterogeneity	
					*P* value	*I* ^2^
Serum prolactin increased^a^	4 [11, 16, 25]	1709	1.59; 0.79–3.21	0.190	0.120	40%
Alanine aminotransferase increased	4 [11, 16, 25]	1709	1.42; 0.82–2.47	0.210	0.710	0%
Triglycerides increased	3 [16, 25]	1667	0.89; 0.69–1.14	0.360	0.810	0%
*γ*-glutamyltransferase increased	3 [16, 25]	1667	1.01; 0.66–1.54	0.970	0.630	0%
White blood cell count increased	1 [25]	892	0.65; 0.34–1.27	0.210	NA	NA
Serum bilirubin increased	1 [25]	892	1.04; 0.55–1.95	0.910	NA	NA
Constipation	2 [16]	775	0.99; 0.34–2.92	0.980	0.620	0%
Diarrhoea	3 [16, 25]	1667	1.34; 0.81–2.23	0.250	0.860	0%
Nasopharyngitis	1 [25]	892	0.93; 0.61–1.42	0.750	NA	NA

^a^Mantel-Haenszel random-effects model.

CI, confidential interval; FD, functional dyspepsia; NA, not applicable; and RR, risk ratio.

## Abbreviations

CI: Confidential interval
EPS: Epigastric pain syndrome
FD: Functional dyspepsia
OTE: Overall treatment efficacy
PDS: Postprandial distress syndrome
PGSI: Patient's global symptomatic improvement
RCT: Randomized controlled trials
RR: Risk ratio
tid: Three times daily.
